# HOST-MICROBE METABOLIC INTERACTIONS IN DISEASE AND AGING

**DOI:** 10.1093/geroni/igad104.0596

**Published:** 2023-12-21

**Authors:** Xiaoling Li

**Affiliations:** NIEHS, Research Triangle Park, North Carolina, United States

## Abstract

As a key mediator of environmental influences on cellular functions, metabolism is crucial for cellular adaptation to the environment. Perturbations of metabolic homeostasis by various environmental factors, including diet and lifestyle, are associated with numerous metabolic diseases and contribute to acceleration of aging. To understand how metabolism mediates environmental regulation of cellular functions and how disruption of metabolic homeostasis contributes to pathogenesis of diseases and aging, we have been focused on a nutrient-sensing pathway consisting of NAD+, SIRT1, the most conserved mammalian NAD+-dependent protein deacetylase/deacylase, and protein acylations. Employing genetically modified mice and cultured cells as model systems, we have recently shown that NAD+ and SIRT1 are key mediators of host-microbe metabolic interactions. On one hand, host NAD metabolism is regulated by gut microbiota. On the other hand, SIRT1 age-dependently modulates gut microbiota through regulation of intestinal bile acid metabolism and Paneth cell activation. Furthermore, we identified SIRT1 as a key regulator of methionine metabolism in embryonic stem cells and developing embryos, thereby modulating their sensitivity to methionine restriction, a dietary regimen that protects against metabolic diseases and aging. Intriguingly, we recently demonstrated that dietary methionine restriction impairs anti-tumor immunity through interaction with gut-microbiota. Together, our findings uncover an important role of the NAD+-SIRT1 axis in mediating host-microbe metabolic interactions that influence disease and aging.

